# Kate Mountstephen

**Published:** 1887-09-17

**Authors:** C. G. Furley


### Kate Mountstephen.

By C. G. Furley.

(Continued from p. 404, vol. ii.)

### Chapter IV.—Sister Katherine.

"You have broken with Harry!" exclaimed Mrs. Jeffreys a few hours later, when, Harry being gone, Kate came to explain that her brief engagement was at an end. "Are you mad? Don't you know that you will never again have such a chance? Why did you do it?"

Kate told of Evelyn Ellis, and her letter.

"Oh! you goose, you idiot! I don't know which of the two is the most stupid, you or Harry. Everyone in London knew of the accident to Mr. Barnett's child forty-eight hours before you sent for me to come here and chaperon you while Harry was in the house. It was in all the evening papers—quite a sensational paragraph—and every one who knew the Barnetts at all was aware that the loss of his baby had caused the old man to take a shock of paralysis, and that nothing short of a miracle could save him. Depend upon it, Miss Ellis was aware of the state of affairs when she wrote, though, like a shrewd woman—a woman who guessed that his fancy for her might be over—she did not allude to the possible change in his position, but assumed that he was as much in love with her as ever. Oh! she is clever! as clever as you are stupid! But can nothing be done? Can't I interfere."

"No," said Kate, "nothing can be done. I have no doubt that he is with her now, and has renewed his engagement; and even if the accusation you bring against her were more than a matter of mere suspicion—and it is not—it is not our place to interfere with a man in a matter where his honour is concerned."

Sister Katherine is tall, and slim, and pale, but her pallor in no way indicates any feebleness of health, and her slender figure has all the lissome grace which used to mark Kate Mountstephen when she rode to hounds. Yet she is changed in some degree. One straight deep line between the eyebrows suggests a trouble which has been fought with and conquered; the fearless grey eyes look out more softly on the world than they used to do; and the firm, calm mouth shows more flexibility and sensitiveness in its curves. But the smile is as bright, the voice as sweet and cheerful, and the hand and heart as ready to help as they were five years ago, when Harry Barnett demanded her care.

It was strange, it seemed strange even to Kate herself, that the episode of his love, an acquaintance which had begun and ended within a month, should have become an important factor in her life. She was not romantic, she told herself; the last woman in the world to fret over lost happiness or disappointed hopes; and yet, in spite of her temperament, in spite of her strenuous efforts to forget her own grief in helping others, in spite of the bitter sorrow of losing her father, which came within a year of her parting with Harry, in spite of everything,—that one brief period of utter happiness had remained the centre of her life, the point to which her thoughts continually turned in those brief moments which she spared to musing on her personal concerns.

It was not, however, love for Harry Barnett that had been the direct influence which had made her leave her home at Coteswold to become a nurse in a London hospital. That had been another lover's work. The gentleman who bought Dr. Mountstephen's practice thought he would like to possess also Dr. Mountstephen's pleasant house and considerable savings. To attain this he made a proposal of marriage to Dr. Mountstephen's daughter, who returned a negative answer too courteous to be other than definitely final. Thereat the suitor's sentiments immediately changed; disappointment developed spite, and he began to harass Kate in the most disagreeable manner he could think of—by interfering with the nursing she still did in the cottages. Finally he wrote her a note requesting her to abstain from any interference with his patients, however well-intentioned, as he could not assume that she was in any way competent to be useful, not being a hospital-trained nurse.

This remark roused Miss Mountstephen's temper. She determined that never again should such a taunt be flung at her; and so let her house, went to a London hospital, and there, having passed through her probation with unusual distinction, she emerged in due time as Sister Katherine.

Her original intention had been to return to Coteswold, armed with her certificate, to crush the new doctor's objections, and work as of old among her own people; but retaliation gets flat when too long delayed, and by the time her studies were over she had lost taste for revenge. She contented herself with paying for a district nurse at Coteswold, and remained in the hospital where she had passed her probation, as Sister Katherine, the head of the children's ward.

"You ought to be happy, I am sure," said one of the nurses to her one day; "I would be content if I were a ward sister."

"I like my post well enough," said Kate; "but it has its responsibilities. You need not envy me."

"But I do, for one thing only. You, having the charge of a ward, are never sent out, as some of us are, to nurse private patients. I have just come back from a case, and oh! Sister Katherine, I was so tired of it. It sounds horrible to say it, but I came near to hating my patient. I suppose I am not fitted for nursing; but, indeed, I never before felt anything but interest and pity for those I had under my care. It was Mrs. Barnett's own fault that I was so indifferent to her. One makes allowance for the caprices of sick people; but I never in my life met anyone so selfish and wilful."

"You said Mrs. Barnett, did you not?" remarked Kate, who had been listening only indifferently till the name was mentioned.

"Yes. Her husband is a brewer—at least he owns a large brewery—and I suppose they are immensely rich. Such a nice man Mr. Barnett seems, too, and very kind to his wife; while she treated him only with rudeness and contempt."

"She was ill," answered Sister Katherine, more to reassure her own sinking heart than with any thought of softening the nurse's feelings.

"I fancy her indifference was not due to illness alone. Her maid, who helped me to nurse her, was present more than once when she treated him with discourtesy, and seemed to think it was nothing unusual. She was never civil to him except when she wanted a cheque from him, the maid told me; and she seemed almost to hate her child, who was the dearest little creature I ever saw, much sweeter than any of your hospital chicks, Sister."

"The hospital chicks haven't had much chance of growing sweet in the homes they come from. But tell me about this one. Is it a boy or a girl, and how old? I am sure it must be lovable; Harry Barnett's child couldn't be otherwise."

"You know Mr. Barnett, then?"

"I knew him some years ago, before his marriage," said Kate, speaking quite calmly, as if Harry Barnett had been a mere acquaintance of former days; but turning her face away from the nurse's gaze, for she knew that the eyes cannot be so well controlled as the voice. "But about the child?" she added.

"Oh, yes! He is a boy, about four years old, with curly brown hair and the merriest blue eyes. It rested me more than anything to go to the nursery and have a romp with him, after his mother had been particularly trying. You would adore him, Sister, you who are fond of children."

"I should like to see him," Kate admitted, and now there was a touch of longing in her tone. "And his mother isn't fond of him, you say."

"She didn't care a straw about him—would never have him brought into her room, and grumbled dreadfully if she heard his voice upon the stair."

"I am sure it was only illness that made her fretful," repeated Kate, determined not to allow a fault in the woman Harry Barnett had married, the woman who, after all, was his first love, and must surely have had some true charm to win him. "Now that she is better she will be fond of both her husband and her child."

"But she isn't better! If she had got well I shouldn't have minded what I said of her; but it is so horrible, so detestable, to feel angry with the dead."

"The dead!"

"Yes, she died last night. It was all her own fault. It was a simple enough case—typhoid, and not very serious; but she would insist on doing everything she should not have done, and that was the end of it. And I know people will think I neglected her in some way, though indeed I did my duty, so far as she would let me." At this point the nurse, worn out by long anxiety and overwork, burst into tears.

"Nurse! Nurse Elliot!" cried Kate, in distress, "don't break down so. You may be quite sure everyone knows you have done your best. You are simply depressed with fatigue, and must lie down for a few hours." And Sister Katherine devoted all her scant leisure that afternoon to soothing Nurse Elliot till she slept.

She was glad of the occupation to keep her mind from dwelling on the news that had come, like a pebble dropped into smooth waters, to disturb the calm of her resignation. And yet the thought would not by any means be put away. Through all she did that day ran the knowledge that Harry Barnett's wife was dead, and that this death might bring a change to her life. The dull, grey path of duty was broken by strange visions of pleasant restful meadows; only mirage perhaps, but perhaps foreshadowings of happiness to come; and when that evening she essayed to tell a fairy tale to one of her little patients it would run into a narrative of how a prince was kept in prison by the magic of a cruel sorceress, yet at last gained his freedom, and married the princess who had been waiting faithfully for him for years and years.

"What am I thinking of?" she asked herself, when at last she was alone in her little bare room, and had nothing to help her to disguise from herself the current of her dreams. "May God forgive me for indulging in such fancies—cherishing selfish hopes beside an open grave!"

And yet it seemed to her as if Harry Barnett must needs enter into her life again, and day after day for many weeks she looked for some sign from him. But none came. Whether he could not find her or would not seek her she did not know; but it seemed to her that it must be the latter. She still communicated occasionally with some of the Coteswold people; if Mr. Barnett inquired there he would easily learn where she was.

"No, he doesn't want to find me!" she said to herself at last, when hope had sunk to a shamed despair. "After all, what right had I to think he would? He had known me for only a month when we parted, and his love was more than half gratitude. And men are not like women: they can love a score of times with equal fervour. I came after the woman whom he married; how can I tell who will succeed me?"

So the brief bright dream, intoxicating in its evanescent sweetness, was again put away, and Sister Katherine devoted herself more strenuously than ever to the work that lay to her hand. But she was quieter than she used to be; a touch of sadness was added to her calm. Life had been in one sense hopeless enough after she had parted from her lover; but it had not been borne down by that sense of absolute desolation which weighed upon her now that she believed he had not cared to seek her again when he was free to do so. She could bear bravely the heaviest blows of circumstance; but to feel herself wholly unloved and undesired was bitter exceedingly.

"Happily there are the children," she said to herself; "they always seem to want me." And though the child-patients in the hospital were not by any means the most attractive specimens of youthful humanity one could meet with—untaught little City arabs most of them, rendered still more selfish and fretful by pain and weakness—they helped Kate to bear her burden. Their needs left her less time to think of private griefs.

"I think they are more lovable for being so ignorant and neglected," she said once to one of her probationers, who complained of her charges being destitute of the first instincts of gratitude and honesty; "I always love those people best who demand most from me."

Nevertheless, when one day a child of a different stamp was brought in, a boy between four and five years of age, clean and daintily clad, evidently of another class than most of the patients, she could not refuse to receive him, though the ward was already crowded. He was brought by a policeman, who had picked him up after he had been run over by a cab.

"He can't give a very coherent account of hisself, poor little chap," said the policeman; "but so far as I can make out he's run away from his nurse and lost his way trying to find her again; and then got mixed up with those cabs and carriages and got run over. His leg seems to me to be broken, and he's pretty badly bruised, so I thought this was the place to bring him to."

"I'm afraid we can't receive him," said the matron, to whom this explanation was addressed. "There is not an empty bed in the whole hospital."

"Don't refuse to take him in," interposed Sister Katherine. "I can put him up somehow; I don't mind giving him my own bed."

"And sleeping on the floor yourself, Sister?" answered the matron.

"There's no need for that; I have a sofa," replied Kate, with a smile; "but even if there were, I want this little fellow; I should be quite grieved if he were turned away."

"As you like, Sister," said the matron; and the child was taken into Kate's room, a tiny slip of an apartment with a window looking into nothing more cheerful than a paved courtyard, surrounded by iron-barred kitchen windows. The children's ward itself looked out on a strip of garden that lay between the hospital and the street; but the ward-sister was less favoured than her patients, and did not complain because her room was placed at the dreariest and most unattainable end of a long corridor. Here she had arranged a few treasures from the old house at Coteswold, so to her the place had a homelike look that cheered her when she was weary or sad. Here the new patient was brought and made as comfortable as splints and bandages would permit. At first he cried pitifully enough, but Sister Katherine spent with him all the time she could spare from the large ward, and at last succeeded in soothing him, and eliciting from him all he knew of himself.

It was not much. His name was "Frank," he said, but he could not tell his surname, nor where he lived. One piece of information he did impart, however, which Kate looked upon as mythical. "I have seen you before," he told her; and when she expressed some doubt as to this statement, he explained that his papa had a portrait of her which he had shown to him several times, telling him that this was a lady who had been very good to him, and that he wanted to see her again, but could not find her. Kate smiled, but thought the child was talking nonsense. Nevertheless, she felt drawn to this unknown Frank more than the mere fact of his being a pretty boy would account for. She hovered around him at every spare moment, and sat up far into the night telling him stories, and singing soft cradle-songs to make him forget the pain and fever of his injuries, feeling herself well repaid when he stretched out his arms to her, saying, "I likes you. I shall tell papa to come here to find you."

"If your papa himself ever finds you, poor little man," said Kate to herself.

(*To be continued.*)

